# Modulators of γ-Secretase Activity Can Facilitate the Toxic Side-Effects and Pathogenesis of Alzheimer's Disease

**DOI:** 10.1371/journal.pone.0050759

**Published:** 2013-01-07

**Authors:** Željko M. Svedružić, Katarina Popović, Vesna Šendula-Jengić

**Affiliations:** 1 Medical Biochemistry, PB Rab, Faculty of Medicine, University of Rijeka, Rab, Croatia; 2 Department of Biotechnology, University of Rijeka, Rijeka, Croatia; 3 Neurology and Geriatrics, PB Rab, Faculty of Medicine, University of Rijeka, Rab, Croatia; Torrey Pines Institute for Molecular Studies, United States of America

## Abstract

**Background:**

Selective modulation of different Aβ products of an intramembrane protease γ-secretase, could be the most promising strategy for development of effective therapies for Alzheimer's disease. We describe how different drug-candidates can modulate γ-secretase activity in cells, by studying how DAPT affects changes in γ-secretase activity caused by gradual increase in Aβ metabolism.

**Results:**

Aβ 1–40 secretion in the presence of DAPT shows biphasic activation-inhibition dose-response curves. The biphasic mechanism is a result of modulation of γ-secretase activity by multiple substrate and inhibitor molecules that can bind to the enzyme simultaneously. The activation is due to an increase in γ-secretase's kinetic affinity for its substrate, which can make the enzyme increasingly more saturated with otherwise sub-saturating substrate. The noncompetitive inhibition that prevails at the saturating substrate can decrease the maximal activity. The synergistic activation-inhibition effects can drastically reduce γ-secretase's capacity to process its physiological substrates. This reduction makes the biphasic inhibitors exceptionally prone to the toxic side-effects and potentially pathogenic. Without the modulation, γ-secretase activity on it physiological substrate in cells is only 14% of its maximal activity, and far below the saturation.

**Significance:**

Presented mechanism can explain why moderate inhibition of γ-secretase cannot lead to effective therapies, the pharmacodynamics of Aβ-rebound phenomenon, and recent failures of the major drug-candidates such as semagacestat. Novel improved drug-candidates can be prepared from competitive inhibitors that can bind to different sites on γ-secretase simultaneously. Our quantitative analysis of the catalytic capacity can facilitate the future studies of the therapeutic potential of γ-secretase and the pathogenic changes in Aβ metabolism.

## Introduction

Alzheimer's disease is a slowly progressing neurodegenerative disorder with a fatal outcome [1], [2]. Symptomatic therapies can provide only a modest temporally relief, and the death occurs after a prolonged hospitalization as a result of debilitating loss of the brain functions [1], [2]. Large efforts in basic and pharmaceutical research are steadily providing diverse therapeutic strategies and potential targets [1], [3]–[5]. Some of the therapeutic approaches have reached clinical trials, including the phase III [1]. Unfortunately, none of the clinical trials have led to effective therapies due to lack of desired effects or due to unacceptable toxic side-effects [1]. The repetitive failures of diverse therapeutic approaches show that we still lack some key insights into molecular mechanism behind this complex disease.

Main target of the current drug-development efforts is a membrane embedded aspartic protease, γ-secretase [1], [3]–[5]. γ-Secretase is composed of four subunits: Aph1, Pen2, glycosylated nicastrin, and endo-proteolyzed presenilin as the catalytic core [6]. γ-Secretase has more than 50 different physiological substrates, some of them participate in vital cell-signaling pathways [6]. Alzheimer's disease is a result of poorly understood changes in γ-secretase's activity on transmembrane section of 99-amino-acids-long C-terminal fragment of amyloid precursor protein (C99-APP or just C99) [6]. The C99 substrate is cleaved in two different peptides. Hydrophilic C-terminal AICD fragment is cleaved first, than the remaining hydrophobic N-terminal fragment is cleaved in a series of processive steps that give Aβ peptides varying in length from 1–37 to 1–49 [7]–[9]. The pathogenesis is usually attributed to different processes that lead to decrease in Aβ 1–40 production and increase in production of the longer more hydrophobic Aβ peptides [10], [11]. The later can readily aggregate and trigger still unknown sequence of neurotoxic events [10], [11].

A large number of structurally diverse γ-secretase inhibitors have been prepared [3]–[5]. They are usually classified according to their structures, since a classification according to the mechanism of action, or the binding site, is still an open challenge [3]–[5]. Transition state inhibitors, that target the active site aspartates, have been prepared with specific modifications from previously known inhibitors of aspartic proteases [12], [13]. DAPT, compound E, LY-411,575 and LY-450,139 (semagacestat) are a group of inhibitors with very similar structures and functional properties, and still poorly understood mechanism of action [14]–[18]. Most likely they all bind at the C-terminal section of transmembrane segment 7 in presenilin 1, which could be in proximity to the substrate-docking cavity and the active site aspartates [5]. Aryl-sulfonamide and aryl-sulfone inhibitors can readily disrupt the γ-secretase-DAPT interaction and therefore could share very similar mechanism of action [17]. NSAID inhibitors and their derivatives are a diverse group of inhibitors that target presenilin 1 and C99 substrate [19]. The inhibitors that target C99 substrate have weak potency, and possibly could interfere with potentially pathogenic substrate dimerization [20]. However those interactions lack the specificity and could not be used for development of promising drug candidates [21].

A considerable number of γ-secretase inhibitors have very impressive nanomolar and even picomolar IC50 values, however none of the inhibitors has shown clinical efficacy [3]–[5]. This clearly indicates that the current strategies based on development of highly potent inhibitors with diverse structures cannot be successful without adequate mechanistic insights. The toxic side-effects are usually attributed to interference with numerous physiological processes that depend on γ-secretase, most notably the Notch signaling pathway [1], [3]–[5]. Thus, the current drug-design efforts are concentrated on development of small-molecules that can modulate production of different Aβ products without full inhibition of γ-secretase activity, i.e. γ-secretase modulators as opposed to γ-secretase inhibitors (GSM *vs*. GSI). Such strategy is supported by recent studies which showed that the pathogenic changes in Aβ products can come with significantly different changes in the enzyme turnover rates and AICD production [10], [22], [23].

The frequent definition of γ-secretase modulators as the compounds that can change ratio between Aβ 1–38, Aβ 1–40, Aβ 1–42 production while sparing Notch activity, is inadequate and therefore often confusing [7], [10], [11], [19], [24], [25]. This definition does not take into account the complete catalytic mechanisms of γ-secretase, and many of the important features exhibited by its inhibitors [7]–[11], [14], [17], [24], [26]–[30]. Also none of the published studies, used assays that could differentiate between the actual modulation of the catalytic mechanism of γ-secretase, from the inherent differences caused by different Michaelis-Menten constants for different catalytic products [8], [10], [31], [32]. We have to develop novel screening strategies, which can incorporate diverse factors that affect Aβ production and its inhibition into one workable molecular mechanism. In general in enzymology, modulators of an enzyme activity and the underlining mechanism, can be revealed by studying changes in the enzyme activity caused by gradual increase in the substrate levels until full saturation is achieved ([33], or pp. 289–294 in ref. [31], or p. 251 in [34]). Such approach is very likely to give insightful results with γ-secretase, since it is well-documented that gradual saturation of γ-secretase with its substrate can affect its interaction with different inhibitors and its different Aβ products [8], [10], [14], [26]–[29], [32], [35], [36]. Furthermore, diverse studies on humans, experimental animals, cells and enzymes showed a strong correlation between increase in γ-secretase saturation with its C99 substrate and the potentially pathogenic processes [10], [29], [32], [37]–[49].

γ-Secretase can bind at the same time its substrate, its transition state inhibitor, and its different non-transition-state inhibitors [17], [26]–[30], [35], [36], [50]. These observations lead to a proposal that different molecules can bind to γ-secretase at different sites. The transition state inhibitors can bind to the active site aspartates in the central aqueous cavity [10], [26]–[28], [51]. Hydrophobic substrate can dock into hydrophobic enzyme interior [10], [28]–[30], [50], [52], while different non-transition state inhibitors could bind to poorly defined allosteric site on presenilin 1 [17], [26]–[30], [50]–[52]. Interestingly, the binding of transition state inhibitors and different alleged allosteric inhibitors, can be affected by the substrate or other inhibitors at higher concentrations [14], [17], [26]–[28], [30], [35], [36], [53]. This indicates that γ-secretase can bind its substrate and its inhibitors to different sites at different concentrations, and that different sites have different functional properties [10], [26]–[30], [35], [36]. Subsequent more quantitative analysis of different γ-secretase inhibitors, showed that changes in the substrate level can change dose-response curves and the inhibition potency by more than one order of magnitude [14]. At lower substrate levels different inhibitors show unusual biphasic activation-inhibition dose-response curves for Aβ 1–40 and Aβ 1–42, that can change to standard dose-response curves at the higher substrate levels [9], [14]. Interestingly, the biphasic dose-response can be observed only in the pathogenic β-secretase → γ-secretase branch of APP metabolism, while the α-secretase → γ-secretase branch shows a standard dose-response [14].

These unusual biphasic dose-response curves can be observed with semagacestat (LY-450,139) [15], [16], DAPT [9], [14], [54], but also with transition state inhibitors [14], and sulfonamide inhibitor avagacestat (BMS-708,163) [16], [55]. Thus, these unusual biphasic curves might be an inherent feature in the enzymatic mechanism of γ-secretase. The biphasic inhibition could also cause potentially toxic Aβ-rebound in experimental animals and humans [14]–[16], [55]. Phase III clinical trials showed that biphasic inhibitor semagacestat (LY-450,139) can irreversibly advance cognitive decline relative to the placebo group in addition to the toxic side-effects that can be attributed to interference with the physiological functions of γ-secretase [56]. Dose dependent cognitive decline was also observed in phase II clinical studies with another biphasic inhibitor avagacestat [55], [57].

In this study, we provide a comprehensive conceptual and numerical description of modulation of γ-secretase activity by biphasic inhibitors using one of the most frequently analyzed inhibitors, DAPT [9], [14], [17], [51]. The principal advantage of our study is that we are not only looking how drug-candidate affects γ-secretase activity, but also how it affects γ-secretase's ability to respond to changes in Aβ metabolism. This shift in focus is crucial for development of novel therapeutic approaches, since considerable experimental evidences indicated that changes in APP metabolism can be the common pathogenic process in different alleged causes of Alzheimer's disease [8], [10], [32], [37]–[49], [58]. The presented molecular mechanism could explain the failure of the previous drug-candidates. Alternative drug-development strategies are described in conclusion, together with a discussion how presented experiments can facilitate the future evaluation of the therapeutic potential of γ-secretase.

## Results

### γ-Secretase shows biphasic “activation-inhibition” dose-response curves with DAPT: description of the basic parameters (Fig. 1)

**Figure 1:**
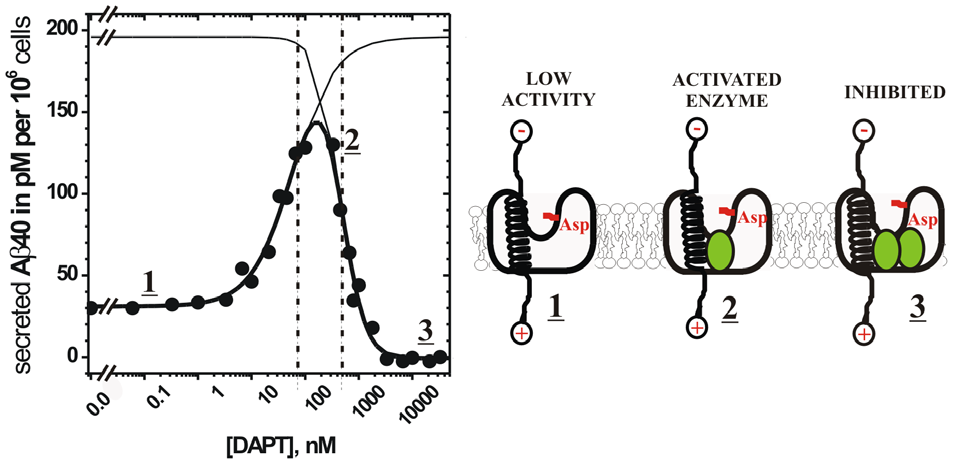
γ-Secretase activity in HeLa cells shows biphasic “activation-inhibition” dose-response curves in the presence of DAPT **[17]**, **[18**] **.** Modulation of γ-secretase activity by DAPT was measured by following secretion of Aβ 1–40 from HeLa cells using Aβ 1–40 specific ELISA. The observed biphasic dose-response curves can be described numerically using nonlinear regression and the equation 1 (Table 1). The calculated parameters allow tracing of two binding events, which can be separated mathematically as the activation phase and the inhibition phase (thin lines), with the corresponding EC50 and IC50 values (the vertical dashed lines). Different phases in the biphasic dose-response curve are marked with underlined numbers and the corresponding molecular interactions are illustrated schematically (C99 substrate can be shown as a transmembrane helix [75], while γ-secretase can be shown as a membrane embedded complex with a central aqueous cavity [76]). Complex **1** represents γ-secretase activity on its substrate in the absence of DAPT (31 pM of Aβ 1–40 secreted per 10^6^ cells, Table 1). Complex **2**; DAPT can activate γ-secretase activity on C99 substrate only if both DAPT and the substrate bind to γ-secretase simultaneously. Complex **3**, complete saturation with DAPT leads to inhibition, indicating that there are at least two different binding events for DAPT, one activating and one inhibiting.

We measured dose-response curves for DAPT by following secretion of Aβ 1–40 in cultures of HeLa cells. Similar to the previous studies [9], [14], we find that DAPT exhibits a biphasic “activation-inhibition” dose-response curves. The biphasic dose-response curves must represent at least two different binding events [34], DAPT binding at the activation site and at the inhibition site (Fig. 1). Different binding events can be described numerically using an equation that combines the standard activation and the inhibition functions (eqn. 1). Six free fit parameters can be resolved by nonlinear regression (eqn. 1): initial activity (IA), calculated maximal activity plateau (MA), maximal inhibition (MI), EC50 for activation, IC50 for inhibition, and the corresponding Hill's coefficients (Table 1).

**Table 1:** Best fit parameters for the biphasic activation-inhibition dose-response curves (eqn. 1)^a^.

Initial Activity (IA)	Maximal Activity (MA)	Maximal Inhibition (MI)	Activation EC50 nM	Activation Hill’s coef	Inhibition IC50, nM	Inhibition Hill’s coef.
[2σ CI]^b^	[2σ CI]^b^	[2σ CI]^b^	[2σ CI]^b^	[2σ CI]^b^	[2σ CI]^b^	[2σ CI]^b^
31 ± 4	196 ± 80	0.5 ± -3.4	72 ± 20	1.09 ± 0.4	472 ± 95	1.92 ± 0.5
[23, 36]	[100, 298]	[-5, 7]	[50, 104]	[0.5, 1.7]	[365, 590]	[1.2, 2.7]
31^c^	196 ± 35	0^c^	72 ± 9	1.09 ± 0.16	472 ± 35	1.92 ± 0.25
	[155, 248]		[61, 84]	[0.75, 1.25]	[422, 525]	[1.6, 2.34]

The units for all activity measurements are: Aβ 1-40 secreted in pM per 10^6^ cells.

a the best fit values ± standard error were calculated using nonlinear regression and eqn. 1.

b two sigma confidence intervals as described in methods section.

c the initial activity (IA) and the maximal inhibition (MA) can be measured separately with high confidence, and thus taken as constants in nonlinear regression. This can give significantly sharper two sigma confidence intervals and standard errors, due to increase in the number of degrees of freedom [34].

The biphasic activation-inhibition dose-response curves represent at least three different molecular interactions. In the absence of the inhibitor, there is only the enzyme-substrate complex and its activity is 31 pM of Aβ 1–40 secreted per 10^6^ cells (complex 1, Fig. 1). Gradual increase in DAPT concentration leads to increase in the enzyme activity so that maximal activity of 145 pM Aβ 1–40 secreted per 10^6^ cells can be observed at 108 nM DAPT. DAPT can activate γ-secretase activity on C99 substrate only if both DAPT and C99 can bind to γ-secretase simultaneously (complex 2, Fig. 1). Further increase in DAPT leads to decrease in Aβ 1–40 secretion and ultimately to complete inhibition. This indicates that at higher concentrations DAPT can also bind at the inhibition site (complex 3, Fig. 1).

The calculated EC50 for activation is 72±20 nM, while IC50 for inhibition is 472±95 nM (dashed vertical lines, Fig. 1). Thus, DAPT binds at the activation site with somewhat higher affinity than at the inhibition site, so that the two binding events overlap and can be resolved only mathematically (thin lines, Fig. 1). The calculated plateau for the activation is 196 nM Aβ 1–40 secreted per 10^6^ cells. Thus, if there is binding only at the activation site, DAPT has potential to activate γ-secretase activity by almost 7 fold (i.e. 31 pM vs. 196 pM Aβ 1–40 per 10^6^ cells). Hill's coefficient for the inhibition phase is 1.9±0.5, indicating that more than one DAPT molecule binds to the inhibition site and a cooperative mechanism could be considered. Hill's coefficient for the activation is 1.1±0.2, which indicates that only one molecule binds at the activation site. The two different Hill's coefficients give the biphasic dose-response curves its asymmetric shape (Fig. 1).

The best-fit curve shows a low scatter for data points, which indicates a high precision of the measurements and a good match between the proposed model equation and the experimental data [34]. With a large number of independent data points that are evenly distributed to different parts of dose-response curve, all six free-fit parameters readily converge, and the best fit values can be easily resolved as indicated by the 2σ confidence intervals (Table 1, [34]). The enzyme activity in the absence of inhibitor, and the activity at full inhibition, can be measured directly with high confidence. Thus, setting those values in nonlinear regression as constants can give significantly sharper confidence intervals due to increase in the number of degrees of freedom [34]. The calculated IC50 values are close to the values that were reported in the past [9], [14], [17].

### Saturation of γ-secretase with its C99 substrate competes with the activation by DAPT (Fig. 2)

**Figure 2:**
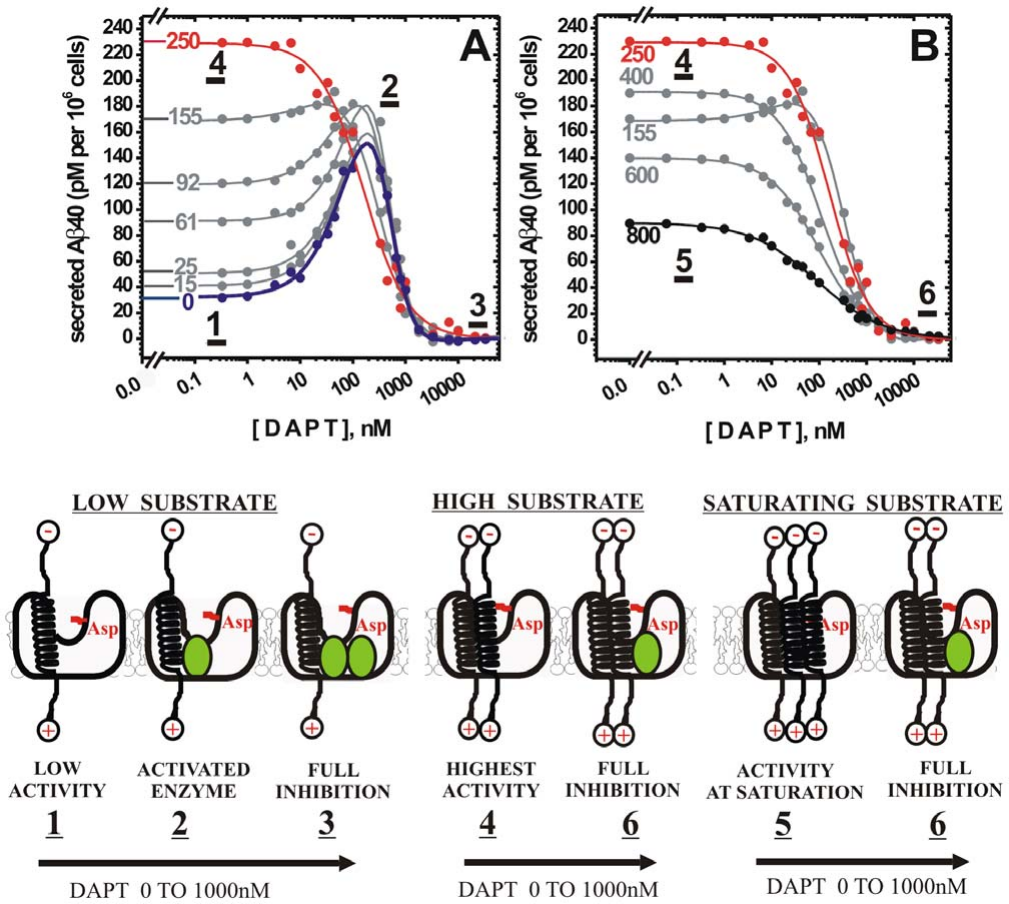
Gradual increase in the extent of γ-secretase saturation with its substrate leads to gradual changes from the biphasic to the standard dose-response curves. Modulation of γ-secretase activity by DAPT was measured by following Aβ 1–40 secretion from HeLa cells using ELISA. The numbers next to each curve indicate pSG5-cDNAwtC99 in ng/mL, the profile at 0 ng/mL represents activity on the endogenous substrate (i.e. untransfected cells just as in Fig. 1). HeLa cells were transfected with increasing concentrations of pSG5-cDNAwtC99 plasmid to achieve gradual increase in expression of C99 substrate and Aβ 1–40 secretion (see methods). The observed profiles can be described numerically using equations 1 and 2, the best-fit values and the corresponding statistic are given in Table 2. (**A**) Different phases in the observed dose-response curves are marked by the underlined numbers to illustrate different molecular interactions schematically. The complexes **1**, **2**, and **3**, represent different interactions between γ-secretase and DAPT at sub-saturating substrate as described in figure 1. A gradual increase in cDNAwtC99 results in a gradual increase in the enzyme activity at the lowest DAPT concentrations, and a decrease in the extent of enzyme activation by DAPT (Table 2). Thus, there is a direct competition between DAPT and the substrate for binding at the activation site (i.e. competition between complex **2** and **4**). This indicates that there are at least two different binding sites for the substrate: the catalytic site and the site that can antagonize binding of DAPT at the activation sites (complex **4**). (**B**) The peak activity is observed at around 250 ng/mL cDNAwtC99, at which point there is no more activation by DAPT, and only inhibition and standard dose-response curves can be observed (i.e. full transition from complex **1** to **4**). Further increase in the substrate (i.e. cDNAwtC99 >250 ng/mL) leads to decrease in Aβ 1–40 secretion (Table 2B), which indicates that the substrate can also bind to the inhibition site (i.e. antagonism between complex **5** and complex **6**).

It has been reported that increase in APP metabolism and the related saturation of γ-secretase with its C99 substrate can abolish its activation by DAPT [14]. Saturating substrate can also interfere with binding of different inhibitors to γ-secretase [17], [26]–[28], [35]. We explore the underling mechanism by measuring biphasic dose-response curves for DAPT in cells that have been exposed to gradually increasing levels of C99 substrate. Gradual increase in C99 expression can be achieved by transfecting HeLa cells with increasing levels of cDNAwtC99 (Fig. 2), using approach that is similar to the previously reported calibration of C99 expression (Fig. 3A in ref. [14]). We find that the transfection with increasing levels of cDNAwtC99 leads to gradual increase in the enzyme activity and gradual changes from the biphasic to the standard dose-response curves (Fig. 2). The full transition is observed at 250 ng/ml of cDNAwtC99, which indicates that the transfection with 250 ng/ml of cDNAwtC99 has the same effect on the dose-response curves for DAPT as the earlier reported shift from the wild-type to the Swedish mutation in APP substrate [14]. We find that gradual increase in cDNAwtC99 concentration leads to a gradual increase in C99 expression and gradual increase in Aβ 1–40 secretion until the maximum of 229±4 pM of Aβ 40 is reached at 250 ng/mL cDNAwtC99 (see methods). Such changes can be expected when γ-secretase activity is measured in cells that have gradually increasing substrate expression [31].

**Figure 3:**
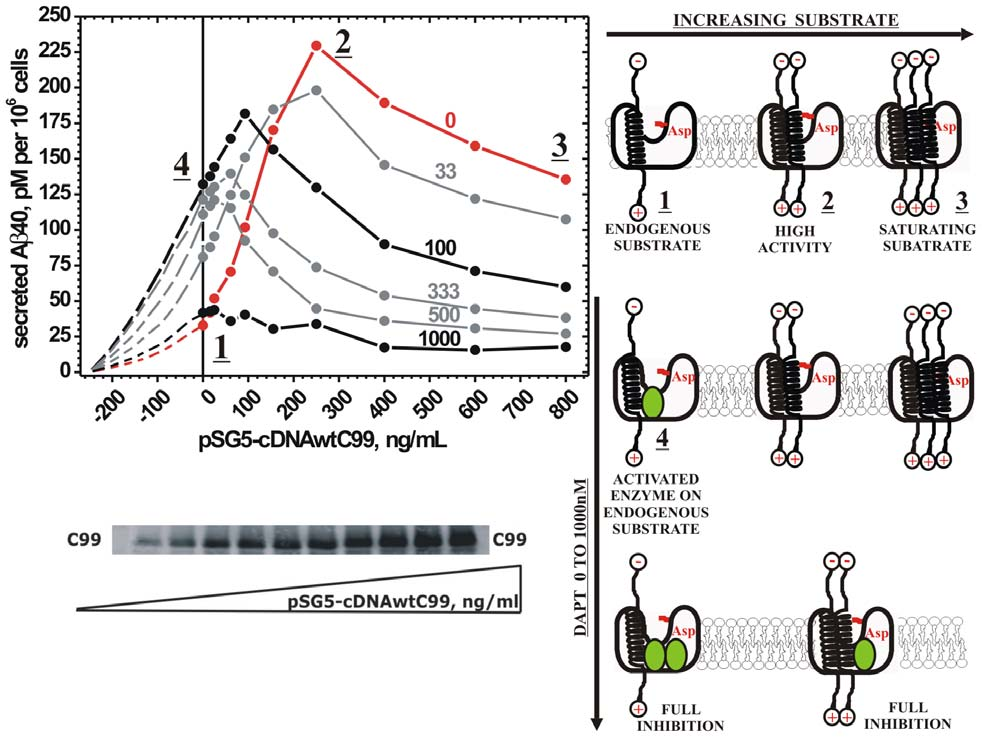
Biphasic inhibitors can reduce γ-secretase's capacity to process its substrates. The molecular mechanism and the physiological significance of the biphasic activation-inhibition dose-response curves can be revealed by re-plotting the data from figures 2 A–B according to the standard approach for studies of modulators of enzyme activity ([33], or pp. 289–294 in ref. [31], or p. 251 in [34]). The Y-axis shows the reaction product, Aβ 1–40 secreted just as in Fig. 2. However the X-axis shows values that are functionally proportional to the extent of γ-secretase saturation with its C99 substrate; i.e. gradually increasing concentrations of cDNAwtC99 that was used to transfect the cells (as shown on the gel strip and described in the methods). The red line represents γ-secretase in the absence of DAPT at different level of saturation with its substrate, i.e. complex **1**, **2** and **3**. Aβ 1–40 secreted at 0 ng/mL cDNAwtC99 and 0 nM DAPT represents activity on the endogenous C99 substrate. Different curves represent different concentrations of DAPT in nM (i.e. activity modulator). Activation by DAPT can induce an apparent shift in the activity profiles to the left of the Y axis (symmetrically extrapolated dashed lines were used to illustrate the shift). This shift indicates that at sub-saturating substrate DAPT can activate γ-secretase by “filling-in” for the subsaturating substrate [31]. At the saturating substrate, DAPT is a noncompetitive inhibitor [31]. These synergistic activation-inhibition effects can drastically reduce γ-secretase's capacity to process it substrate (Table 3). Different sections on the graphs are labeled with the underlined numbers to map the corresponding interactions just as in the figures 1 and 2. Except for complexes **1**, **3** and **4**, this type of analysis cannot clearly resolve the activity range that corresponds to the different interactions as seen in figures 1 and 2.

Just as in figure 1, the different molecular events in figure 2 can be described numerically and the underlining mechanism can be illustrated schematically (Fig. 2 and Table 2). The biphasic dose-response curves for the activity on endogenous substrate (i.e. 0 ng/ml cDNAwtC99) can be described with three different interactions; *i*) the enzyme-substrate complex in the absence of DAPT (Fig. 2, complex 1), *ii*) the enzyme-substrate complex that is activated by DAPT (Fig. 2, complex 2), and *iii*) the enzyme-substrate-DAPT complex that can be inhibited by DAPT (Fig. 2, complex 3). Gradual increase in C99 expression results in gradual increase in the enzyme activity at the lowest DAPT concentrations, but also in decrease in the extent of enzyme activation by DAPT (Fig. 2 and Table 2). For example, gradual increase in cDNAwtC99 from 0 to 92 ng/mL leads to gradual increase in the enzyme activity from 32±3 pM to 119±4 pM of Aβ 1–40 secreted, yet in all cases the calculated maximal activation by DAPT remains to within the experimental error constant and equal to an average value of 189 pM of Aβ 1–40 secreted. Actually, the calculated maximal activation by DAPT is not far from the maximal activity that can be achieved by the increase in substrate expression level (189 pM vs. 229 pM of Aβ 1–40 secreted per 10^6^ cells at 250 ng/ml cDNAwtC99).

**Table 2:** Best fit parameters for the biphasic activation-inhibition dose-response curves with DAPT^a^.

ng/mL of pSG5-cDNAwtC99 used to transfect HeLa cells						
**Data from ** **Fig. 2A** a **:**	**0**	**15**	**25**	**61**	**92**	**Average**
**Initial Activity, IA**	32 ± 3	41 ± 3	51 ± 5	91 ± 6	119 ± 4	n.a. c
**[2σ CI]** b	[27, 37]	[34, 46]	[43, 59]	[81, 99]	[111, 127]	
**Maximal Activity, MA**	217 ± 110	172 ± 90	205 ± 80	180 ± 145	170 ± 95	189
**[2σ CI]** b	[470, 133]	[380, 120]	[510, 121]	[430, 160]	[-, -]	
**Maximal Inhibition, MI**	-1 ± 0.1	0.33 ± 0.7	-0.2 ± 0.1	1.2 ± 0.2	1.9 ± 0.6	0.5 ± 0.2
**[2σ CI]** b	[-1.75, 1.1]	[-1.0, 2.1]	[-1.5, 0.9]	[-1.36, 0.86]	[-2.7, 2.9]	
**Activation EC50, nM**	89 ± 17	71 ± 21	110 ± 34	107 ± 36	178 ± 45	111 ± 41
**[2σ CI]** b	[50, 240]	[105, 243]	[53, 251]	[22, 160]	[-, -]	
**Activation Hill coef.**	1.04 ± 0.2	1.08 ± 0.2	1.0 ± 0.1	1.13 ± 0.6	0.92 ± 0.3	1.0 ± 0.1
**[2σ CI]** b	[0.85, 1.35]	[0.9, 1.45]	[0.9, 1.3]	[0.87, 1.28]	[0.61, 1.5]	
**Inhibition IC50, nM**	407 ± 34	588 ± 35	524 ± 71	489 ± 62	661 ± 80	534 ± 53
**[2σ CI]** b	[479, 724]	[269, 550]	[457, 562]	[150, 602]	[525, 732]	
**Inhibition Hill’s coef**	2 ± 0.3	2.4 ± 0.3	2.2 ± 0.4	2.3 ± 0.5	1.7 ± 0.6	2,1 ± 0.2
**[2σ CI]** b	[1.4, 2.9]	[1.6, 3.13]	[1.6, 2.9]	[1.6, 3.0]	[-, -]	

ng/mL of pSG5-cDNAwtC99 used to transfect HeLa cells					
**Data from Fig. 2B^d^:**	**250**	**400**	**600**	**800**	**Average**
**Initial Activity a IA**	229 ± 4	190 ± 2	140 ± 4	90 ± 1	n.a. c
**[2σ CI]^b^**	[218, 239]	[178, 209]	[128, 159]	[84, 102]	
**Maximal Inhibition a MI**	-0.9 ± 0.4	2.0 ± 0.9	-4 ± 2	2 ± 0.8	-0.5 ± 0.6
**[2σ CI]^b^**	[-3, 1.3]	[-2, 4.3]	[-7, 1.0]	[0.53, 4.25]	
**Inhibition IC50, nM a**	126 ± 9	110 ± 8	98 ± 6	89 ± 5	106 ± 16
**[2σ CI]^b^**	[109, 138]	[99, 118]	[89, 108]	[52, 154]	[67, 230]
**Inhibition Hill’s coef^a^**	1.02 ± 0.07	0.95 ± 0.04	0.84 ± 0.03	0.61 ± 0.07	n.a. c
**[2σ CI]^b^**	[0.89, 1.16]	[0.82, 1.02]	[0.66, 0.96]	[0.53, 0.82]	

The units for all activity measurements are: Aβ 1-40 secreted in pM per 10^6^ cells.

a the best fit values ± standard error were calculated using nonlinear regression and eqn.1.

b two sigma confidence intervals as indicated in methods section [34].

c not applicable, or the corresponding values could not be resolved in the current experiments.

d no activation is observed when DNA concentration is higher than 250 ng/ml. Thus starting at 250 ng/ml the best fit values ± standard error were calculated using a nonlinear regression and eqn.2.

The observed changes in the biphasic curves indicate that there is a direct antagonism between activation of γ-secretase by DAPT and the increase in γ-secretase activity due to increase in the substrate expression level. Therefore, there must be at least two different binding sites for the substrate: the catalytic site (Fig. 2, complex 1) and the second site that can antagonize activation by DAPT (complex 4). When the peak activity is reached at 250 ng/mL cDNAwtC99, there is no more activation by DAPT which indicates that the gradual increase in substrate expression has resulted in a full transition from complex 1 to 4 (Fig. 2). Moreover, once the peak activity is reached further increase in the substrate expression leads to a decrease in Aβ 1–40 secretion (Fig. 2 B). Increase in cDNAwtC99 from 250 ng/mL to 800 ng/mL leads to decrease in Aβ 1–40 secretion from 229±4 pM to 90±1 pM. The decrease in activity indicate that there are at least three different interactions between γ-secretase and its substrate; *i*) the γ-secretase-substrate complex that can be activated by DAPT (Fig. 2, complex 1); *ii*) the γ-secretase-substrate complex that cannot be activated by DAPT due to substrate binding at the second site (Fig. 2, complex 4); *iii*) and the γ-secretase-two-substrates complex that is inhibited by binding of the saturating substrate at the substrate inhibition site (Fig. 2, complex 5).

The EC50 and IC50 values and the corresponding Hill's coefficients reflect the gradual transition from complex 1 to complex 4, and finally complex 5 (Fig. 2 and Table 2). In all of the biphasic profiles the EC50 values for activation and the corresponding Hill's coefficients appear to be within experimental error constant with an average value of 111±41 nM and 1.0±0.1 respectively (Table 2). These values indicate that only one DAPT molecule binds at the activation site [34]. On the other hand, the IC50 values for inhibition and the corresponding Hill's coefficient appear to be within experimental error constant with an average IC50 value of 534±53 nM and 2.1±0.2 respectively for all biphasic dose-response curves (Table 2). The measured Hill's coefficients are suggesting that more than one DAPT molecule can bind to the inhibition site at the lowest substrate levels and also a cooperative mechanism could be considered [34]. Interestingly, full transition from the biphasic to the standard dose-response curves leads to decrease in the IC50 value to an average value of 106±16 nM. The corresponding Hill's coefficients show a continual decrease from an average value of 2.1±0.2 at the lowest substrate, to 0.61±0.07 at the highest substrate (Table 2). The observed changes indicate that at the higher substrate levels there is some degree of antagonism between the inhibition by DAPT and the inhibition by saturating substrate [34] (Fig. 2, complex 5 vs. complex 6). The presented observations can further strengthen earlier proposals that increase in saturation of γ-secretase with its C99 substrate leads to a relative decrease in Aβ 1–40 production [8], [10], [32], due to modulation of γ-secretase's activity by binding of multiple substrate molecules [10].

### DAPT activates γ-secretase by “filling-in” for the sub-saturating substrate

Physiological significance of the biphasic “activation-inhibition” dose-response curves and the underlining molecular mechanism can be revealed by re-plotting the data from figures 2 A–B according to the standard approach for studies of modulators of an enzyme activity ([33], or pp. 289–294 in ref. [31], or p. 251 in [34]). Just as in the figure 2, the Y-axis in figure 3 shows the enzymatic activity in pM of Aβ 1–40 secreted per 10^6^ cells. However, the X-axis shows values that are functionally proportional to extent of the enzyme saturation with its substrate (i.e. concentrations of cDNAwtC99 used in the transfections). The different curves represent different concentrations of the activity modulator, DAPT, from 0 to 1000 nM.

In general, an enzyme's capacity to process its substrate is defined by the substrate quantity that the enzyme can process before reaching saturation, and by its maximal turnover rates (i.e. the Michaelis-Menten principles [31]). Thus, the re-plots on figure 3 allow quantitative assessment of γ-secretase capacity to process its substrate in cells and its modulation by DAPT (Table 3). The red line in figure 3 shows that in the absence of DAPT an increase in cDNAwtC99 leads to changes in γ-secretase activity that look like a Michaelis-Menten hyperbola for an enzyme that has inhibition at saturating substrate ([33], or pp. 289–294 in ref. [31], or p. 251 in [34]). Precisely, there is a clear functional relationship between the concentration of cDNAwtC99 used in transfection, the expression level of C99 substrate, and the Aβ 1–40 secretion until saturation and the maximal activity are achieved (Fig. 3). The activity at 0 ng/mL cDNAwtC99 and 0 nM DAPT represents γ-secretase activity on its endogenous C99 substrate, and it is equal to 32.3 pM Aβ 1–40 secreted. The maximal activity that can be reached by the transfections is 229.9 pM Aβ 1–40 secreted at 250 ng/mL cDNAwtC99. Thus, γ-secretase activity on its endogenous substrate is only 14% of its maximal activity (Table 3). In another words, γ-secretase in cells has enough catalytic capacity to process its C99 substrate in an equivalent of 250 ng/mL of cDNAwtC99 before it is saturated.

**Table 3:** Specified numerical values from the data shown in Fig. 3.^a^

DAPT, nM	activity on Endogenous substrate ^b^ (Aβ 1-40 secreted in pM per 10^6^ cells)	Maximal Activity that can be achieved by transfection ^c^ (Aβ 1-40 secreted in pM per 10^6^ cells)	Endogenous activity as percentage of maximal activity achieved by transfection d	cDNAwtC99 required to reach maximal activity ^e^ (ng/mL)	cDNAwtC99 required to reach activation by DAPT^f^ (ng/mL)
0	32.4	229.9	14.1	250.0	-
33	81.1	204.5	39.7	191.0	70
100	132.0	182.0	72.5	92.7	120
333	129.0	140.0	92.1	59.8	118
500	111.6	125.0	89.3	39.2	100
1000	43.0	44.7	96.2	14.0	16

a the table shows specified numerical values from the data shown in Fig. 3.

b. Aβ 1-40 secretion activity in untransfected cells at the specified DAPT concentrations (i.e. activation at 0 ng/ml cDNAwtC99).

c. maximal Aβ 1-40 secretion activity that can be achieved by the transfections at the specified DAPT concentrations.

d percentage ratio between the activity on endogenous substrate and the maximal activity that can be reached by transfections (i.e. the percentage ration between values in columns 2 and 3).

e. cDNAwtC99 in ng/mL required for transfection to reach maximal Aβ 1-40 secretion activity at the specified DAPT concentration.

f. cDNAwtC99 in ng/mL that is required in transfections, to reach the same levels of Aβ 1-40 secretion activity as the activation of Aβ 1-40 secretion on endogenous C99 substrate at the specified DAPT concentration.

The re-plots in figure 3 show that DAPT can modulate γ-secretase activity by combining some of the well-known activation and inhibition mechanisms that are described in textbooks ([33], or pp. 289–294 in ref. [31], or p. 251 in [34]). The activity profiles with DAPT have the same Michaelis-Menten shape as the profile without DAPT, just that the DAPT profiles look shifted to the left of Y axis (Fig. 3, the extrapolated dashed lines were used to illustrate the shift). This apparent shift shows that DAPT induced increase in γ-secretase activity at the sub-saturating substrate is due to increase in the enzyme-substrate kinetic affinity (usually described as increase in kcat/Km value pp. 349–377, and pp. 289–294 in [31], and p. 251 in [34]). Thus at the low substrate levels, DAPT behaves as a surrogate-substrate that can “fill-in” for the sub-saturating substrate. At saturating substrate DAPT behaves as a noncompetitive inhibitor (Fig. 3). DAPT can never increase the maximal possible enzyme activity relative to the activity in the absence of DAPT, since the activation and the inhibition phases overlap (Fig. 1).

Both, the activation and the inhibition by DAPT can facilitate decrease in γ-secretase capacity to process its substrates so that potentially harmful saturation can be reached even at otherwise sub-saturating substrate (Table 3). For example, DAPT at 100 nM can activate Aβ 1–40 secretion on endogenous substrate from 32.1 pM to 132 pM, and thus make the enzyme as active on its endogenous substrate as transfection with 120 ng/mL cDNAwtC99 in the absence of DAPT (Table 3). Consequently, there is a decrease in the substrate levels that γ-secretase can process before reaching the saturation from an equivalent of 250 ng/ml to 92 ng/ml of cDNAwtC99 (Table 3). Moreover, due to noncompetitive inhibition there is also a decrease in the maximal activity at the saturating substrate from 229 pM to 182 pM of Aβ 1–40 secreted. Thus, the activation and the inhibition processes have synergistic effect on the decrease in γ-secretase catalytic capacity. Therefore in the presence of 100 nM DAPT γ-secretase activity on its endogenous substrate is as much as 72% of the maximal possible activity (Table 3). At 333 nM DAPT, γ-secretase activity on the endogenous substrate is still activated relative to activity at 0 nM DAPT (Fig. 3). However at 333 nM DAPT the activity on endogenous substrate is as high at 92% of the maximal possible activity, and the enzyme saturation can be achieved with only 92 ng/ml cDNAwtC99 (Table 3). Even at 33 nM DAPT, when there is only a mild activation at the sub-saturating substrate and only mild inhibition at the saturating substrate, the enzyme activity on the endogenous substrate is as much as 40% of the maximal activity, and the substrate levels that the enzyme can process before reaching the saturation have decreased to 192 ng/ml of cDNAwtC99 (Table 3).

Increase in DAPT concentration beyond 333 nM will lead to a situation where activity on the endogenous substrate is almost equal to the maximal possible activity. In those conditions transfections with cDNAwtC99 do not lead to increase in the Aβ 1–40 secretion, which indicates that in those conditions γ-secretase has a very limited capacity to process its different physiological substrates. In summary, we find that DAPT can drastically reduce γ-secretase capacity to process its APP and other physiological substrates. The drastic reduction in the catalytic capacity exists even when γ-secretase is activated DAPT.

### Building model mechanism for γ-secretase interaction with its inhibitors

**Figure 4:**
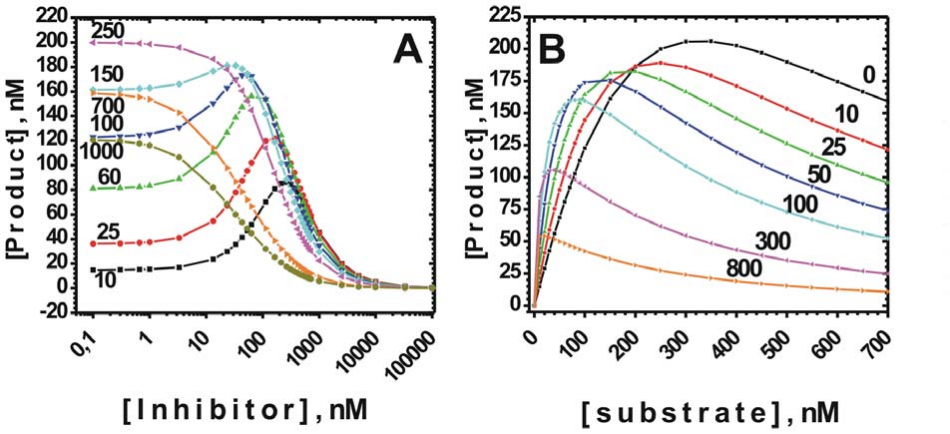
The biphasic dose-response curves (A) and the corresponding enzyme saturation profiles (B) can be modeled using numerical simulation. The panels represent an attempt to simulate experimental data from Fig. 2 and Fig. 3, using the equation that can describe the proposed model mechanism (eqn. 3) and standard MS Excel program. In panel A the X axis shows inhibitor concentrations and different curves represent different substrate concentrations, the opposite combination is used to generate panel B. For both panels Vmax1 = 450, Vmax = 50, Kia = 15 nM, Kii = 120 nM, K0.5s = 300 nM, Ks2 = 500 nM.

The presented description of the biphasic dose-response curves can be used to build a model mechanism for computational simulation of the experimental data (Fig. S1 and eqn. 3). Such simulations can evaluate validity of the proposed model based on overlap between the experimental and the computer generated data. Well-matched computational model can provide a basis for future QSAR studies, and selection of the best drug-development strategies. The equation 3 is the simplest description of the proposed mechanism and its experimental features (Fig. 4).

We find very strong similarities between the experimental data (Figs. 2 and 3) and the computer generated data (Fig. 4 A–B), indicating that the proposed mechanism for biphasic activation-inhibition dose-response curves can be valid. The constants used in simulation were chosen so that the simulated results show the same features as the experimental data, namely: *i*) EC50 and IC50 values are within experimental error close to the experimental values, *ii*) saturation induced shift from the biphasic to the standard dose-response curves leads to a decrease in IC50 value; *iii*) partial inhibition with the saturating substrate; *iv*) the maximal rate observed in the absence of DAPT is higher than the maximal activation by DAPT; *v*) DAPT is a noncompetitive inhibitor at the saturating substrate; *vi*) increase in the extent of the enzyme saturation with its substrate leads to decrease in Hill's coefficient for the inhibition.

Several conditions have to be satisfied to achieve desired overlap between the simulated and the experimental data. First, the activation can be observed only if K_ia_ is lower than K_0.5s_, the higher is K_0.5s_ relative to K_ia_, the more pronounced is the activation. K_ia_ and K_ii_ have to be much lower than EC50 and IC50 values for the activation and the inhibition. The maximal rate for the enzyme in complex with the multiple substrate molecules (Vm2, i.e. Fig. 2 complex 4), has to be only a fraction of the maximal rate for the enzyme acting on a single substrate (Vm1, i.e. Fig. 2 complex 1).

## Discussion

We have described how DAPT can modulate γ-secretase activity in HeLa cells by measuring Aβ 1–40 production in one of the standard assays for studies of modulators of enzyme activity ([33], or pp. 289–294 in ref. [31], or p. 251 in ref. [34]). The new insights are combined in one molecular mechanism (Figs. 1,2,3,4), that is fully consistent with the previous descriptions of γ-secretase interaction with its substrate and its different inhibitors [9], [10], [14]–[17], [26]–[29], [35], [52], [54], [55], [59]. The biphasic dose-response primarily affects potentially pathogenic β-secretase → γ-secretase branch of APP metabolism [14]. Thus, the studies of this phenomenon could offer some key insights in the pathogenesis and novel therapeutic strategies. In coming paragraphs, we will elaborate significance of the four major conclusions that come from this study, namely: *i*) γ-secretase's reaction on its endogenous substrate is not a significant fraction of the total catalytic capacity in cells (Table 3 and Fig. 3); *ii*) the biphasic inhibitors can drastically reduce γ-secretase's capacity to process its physiological substrates and thus facilitate toxic side-effects and possibly pathogenesis (Fig. 3 and Table 3); *iii*) multiple substrate and inhibitor molecules can bind to γ-secretase at the same time and modulate its activity (Fig. 2); *iv*) novel improved inhibitors can be prepared from the compounds that bind to multiple sites on γ-secretase simultaneously. We propose that some of our conclusions on DAPT could be extrapolated to the other biphasic inhibitors of γ-secretase, based on a high degree of numerical and conceptual consistency between our studies and the related studies in the past [9], [10], [14]–[17], [26]–[29], [35], [52], [54], [55], [59].

### Biphasic inhibitors have limited therapeutic potential due to their ability to facilitate toxic side-effects

We find that γ-secretase's activity on its endogenous substrate in cells is far below saturation and its maximal catalytic capacity (Fig. 3 and Table 3). Thus, the molecules of γ-secretase in cells have enough catalytic capacity to process all of their physiological substrates without impeding competition. The biphasic inhibitors have two synergistic features that can severely decrease γ-secretase's capacity to process its physiological substrates (Fig. 3 and Table 3). The activation can make γ-secretase saturated even at otherwise sub-saturating substrate, while the noncompetitive inhibition can decrease the number of active enzyme molecules and therefore the maximal activity (Fig. 3, Table 3 and ref. [31]). This inhibitor-facilitated decrease in γ-secretase's capacity to process its substrate is nicely visible in the earlier studies with H4 cells [16], which showed that even activation of γ-secretase by the biphasic inhibitors leads to a spike increase in accumulation of cellular C99 substrate. The inhibitor-facilitated decrease in γ-secretase's capacity to process its different physiological substrates is a major deficiency, that could make the biphasic inhibitors exceptionally prone to the toxic side-effects [3], [4], [19].

### Biphasic inhibitors could facilitate potentially pathogenic processes

Both, the activation and the noncompetitive inhibition by the biphasic inhibitors could facilitate potentially pathogenic increase in γ-secretase saturation with its C99 substrate (Fig. 3). Different studies on humans, experimental animals, cells and enzymes, showed that a chronic increase in saturation of γ-secretase with its C99 substrate strongly correlates with the pathogenic events [37]–[49]. Some of the examples are: *i*) increased expression of the APP gene [42]–[44], or any other increase in APP metabolism [58], [60]; *ii*) increased activity of β-secretase [37]–[41]; *iii*) or the Swedish mutation in the APP sequence [45], [46]. On the other hand, decrease in the extent of γ-secretase saturation with its C99 substrate can decrease the chances of pathogenesis [61]. Gradual increase in saturation of γ-secretase with its C99 substrate leads to increase in production of the longer more toxic Aβ peptides [8], [10], [32]. Also, increase in C99 substrate above certain threshold could cause potentially toxic disruptions in integrity of cellular membranes [16], [58], [62]–[65]. The noncompetitive inhibition can also produce potentially pathogenic increase in cellular C99 levels [16], due to decrease in the maximal catalytic rates (Fig. 3). Different genetic studies showed that a decrease in γ-secretase activity in cells could lead to potentially pathogenic events [47], [49], while the opposite effects can be observed when γ-secretase expression is increased [48]. In addition, a decrease in the maximal turnover rate is a common feature shared by the noncompetitive inhibitors and different FAD mutations [10], [23], [66].

In summary, the biphasic inhibitors could facilitate potentially pathogenic processes that could explain why clinical trials showed dose dependent cognitive decline with avagacestat [55], [57], and irreversible cognitive decline with semagacestat [56]. On the optimistic side, studies of the biphasic inhibitors could give valuable insights in the molecular pathogenesis and lead to novel drug development strategies.

### The biphasic inhibitors can explain unusual pharmacological properties of γ-secretase inhibition

The Aβ-rebound observed in cells, experimental animals, and humans is an expected pharmacodynamic feature for all biphasic inhibitors [9], [14]–[16], [54], [55]. The inhibitor-induced restriction in the enzyme capacity to process its substrate can explain an apparently absurd observation, that activation of γ-secretase with biphasic inhibitors in H4 cells leads to a spike increase in accumulation of cellular C99 substrate [16]. The biphasic mechanism could also explain why even a moderate inhibition of γ-secretase cannot result in an effective therapy. Moderate inhibition has been proposed several times, based on ongoing debate that there is only about 30% increase in γ-secretase activity in the disease [3], [67]. It is very likely that a 30% decrease in γ-secretase activity would not cause toxic interference with the processing of its physiological substrates. More than a 30% decrease in γ-secretase activity can be expected in heterozygous cells carrying some of the FAD mutations [10], [23], [66], and yet none of those mutants shows significantly toxic disturbance of the Notch signaling pathway. Our results show that a moderate inhibition, and even activation by the biphasic inhibitors can results in a drastic decrease in γ-secretase's capacity to process its physiological substrate (Fig. 3 and Table 3). Thus, it is highly unlikely that even a moderate inhibition by the biphasic inhibitors can lead to a successful therapy.

The presented mechanism indicates that more potent biphasic inhibitors can produce larger decrease in γ-secretase's capacity to process its physiological substrates (Fig. 4), and therefore the acute toxic side-effects that can be readily apparent. The less potent inhibitors can preserve some of the γ-secretase's capacity to process its physiological substrates (Fig. 4), and thus show no evidences of readily apparent acute toxic side-effects. However, such situation can cause a chronic increase in saturation of γ-secretase with its physiological substrate, and thus make the cells more vulnerable to the pathogenic processes caused by changes in APP metabolism. This proposal is in line with different pharmacological properties of two biphasic inhibitors that have very similar structures and functional properties but different potency; LY-411,575 with IC50 close to 0.2 pM and semagacestat with IC50 close to 20 nM [3]–[5], [16], [19], [68]. LY 411,575 showed toxic side-effects early in preclinical studies, while semagacestat showed good tolerance in preclinical studies and in short phase I and phase II clinical trials [69]. Semagacestat showed the toxic side-effects and irreversible cognitive decline only after prolonged phase III clinical trials [56].

### Biphasic inhibition is additional new evidence that multiple substrate and inhibitor molecules can bind to γ-secretase at the same time

Our results can further strengthen earlier proposals that multiple substrate molecules can bind to γ-secretase at the same time and modulate its activity [10], as well as earlier observations that saturation of γ-secretase with its substrate can affect its interaction with different inhibitors [14], [17], [26]–[29], [35], [52], [59]. It has been suggested that the biphasic inhibition is not a result of allosteric regulation since this type of inhibition can be observed with very different inhibitors, including the transition state inhibitors that target the active site aspartates [14], [70]. In addition, at saturating concentrations both C99 substrate and DAPT can facilitate production of the longer more hydrophobic Aβ products [8]–[10], [54]. Combined together, those observations are the most consistent with the proposal that multiple substrate and inhibitor molecules can interact within the active site cavity [10], and thus modulate dynamic processes that control the stability of γ-secretase-C99 complex and the processive cleavages [10].

The results on figures 1, 2 and 3 show that our ability to detect multiple interactions depends on our ability to design experiments that can differentiate between different interactions (see Fig. S1 for more details). This can explain why some studies failed to detect evidences of multiple interactions. The activation by biphasic inhibitors can be observed in cell-based studies, in experimental animals and in humans [9], [14]–[16], [54], [55]. Depending on the inhibitor's kinetic constants the enzyme-based studies show no activation [10], [14], or a mild activation that is usually ignored (such as inhibition by difluoroketone compounds in ref. [70], or inhibition of the Notch substrate by begacestat in ref. [23], or our unpublished results with sulindac-sulfide). The cell-based and the enzyme-based assays share many of the fine features [10], however the enzyme-based assays have much lower sensitivity and different kinetic constants [10], and thus require orders of magnitude higher C99 concentrations [10], [23]. The higher C99 concentrations can compete with DAPT-induced activation (Fig. 2), and also favor formation of C99 dimers [10]. We suspect that the higher C99 concentrations and C99 dimers can antagonize binding of the inhibitor to the activation site in the enzyme-based assays (Fig. 2, complex 2 vs. complex 4).

In general, the extent of an enzyme saturation with its substrate is always a key variable in inhibition and modulation of the enzyme activity ([33], or pp. 289–294 in ref. [31], or p. 251 in [34]). Thus, in principle it could be possible to resolve many of the previously reported confusions about modulation and inhibition of γ-secretase activity [3], [4], [19], if the extent of γ-secretase saturation with its substrate becomes a controlled experimental variable. Unfortunately only a small fraction of γ-secretase studies recognized this importance [8], [10], [14], [27], [28], [32].

### Impact of biphasic inhibitors on the future drug-design strategies

It is highly unlikely that any of the different biphasic inhibitors can lead to an effective therapy, due to their unique ability to facilitate decrease in γ-secretase's capacity to process its substrates. The best alternative could be competitive inhibitors that can shift the saturation to the higher substrate levels without harmful decrease in γ-secretase's capacity to process its substrates (Fig. S2 and ref. [31]). The inhibitor's potency can regulate competition with the substrate, and thus moderate how γ-secretase responds to potentially pathogenic changes in APP metabolism (Fig. S2). The competitive inhibitors can also provide some selectivity between APP and Notch substrates, or Aβ 1–40 and Aβ 1–42 products, by exploiting the relative differences in Michaelis-Menten constants ([31] and Svedružić *et. al*, manuscript in preparation). The presented results suggest that novel competitive inhibitors, with standard dose-response curves, can be prepared from the compounds that can bind to the activation and the inhibition site at the same time. The first lead for such compounds, could be side-by-side or head-to-tail dimmers of the current compounds that have biphasic dose-response curves (Fig. S2). Such compounds could act as competitive inhibitors since the binding to the inhibition site would depend on competition with the substrate for binding to the activation site (i.e. in figure 2, complex 3 would be in competition with complex 4, and the intermediary complex 2 would not exist). In combination with the earlier site-directed mutagenesis, cross-linking, and cross-competition studies, the presented experiments can help in identification of different binding sites ([17], [26], [27], [51] and Fig. S1).

### Impact of the biphasic inhibitors on our understanding of Alzheimer's disease

The presented quantitative analysis of changes in the catalytic capacity of γ-secretase, can be used for evaluation of the pathogenic potential of different cellular processes that affect Aβ metabolism [10], [58], [71]. For example, different FAD mutations can affect to different degree the catalytic capacity of γ-secretase [10], [23], and the biphasic dose-response curves [9]. Thus, the differences in γ-secretase's capacity to process its substrate could explain the differences in the pathogenic potential of different FAD mutations (Svedružić *et. al*, manuscript in preparation). We already know that the Swedish mutation results in a shift from the biphasic to the standard dose-response curves with a 12 fold increase in Aβ 1-40 secretion activity [14]. Thus, this mutation can saturate the catalytic capacity of γ-secretase. Apart from the FAD mutants, there are other cellular processes that can facilitate potentially pathogenic changes in Aβ metabolism, and thus the catalytic capacity of γ-secretase. For example: changes in cholesterol metabolism, membrane oxidation, different forms of Aph1 subunits, activity of minor GPCR, or different process that can affect the relative ratio between α-secretase, β-secretase and γ-secretase driven APP metabolism [22], [37]–[44], [58], [61], [72], [73].

### Concluding remarks

It is necessary to point out that presented interpretation of biphasic dose-response curves for Aβ 1–40 secretion activity does not depend on the transfection efficiency and its experimental variability. It is easy to show that increase in cDNAwtC99 concentration leads to increase in C99 expression and increase in Aβ 1–40 secretion, which indicates that those three process are functionally related (Figs. 2–3 and ref. [14]). In fact, transfection with cDNAwtC99 has the same effect on the biphasic dose-response curves for Aβ 1–40 secretion as the Swedish mutation in APP sequence [14]. The transfection efficacy can change the ratio between the amount of cDNAwtC99 used and the intensity of C99 expression, but not the fact that changes in cDNAwtC99 concentration lead to changes in C99 expression and Aβ 1–40 secretion. Furthermore, we are simultaneously comparing combined results from a large number of different transformation events by looking at the differences between cells treated with different levels of DAPT. Thus, our conclusions are not based on a single transformation event, but on a combined response from full spectra of transformation events, from the lowest to the highest DNA concentration, from the lowest to the highest DAPT concentration. The experimental variability in transfection efficiency can affect the scatter between different data points (Figs. 1,2,3), but not conclusions based on comparisons of relative changes in Aβ 1–40 secretion for the cells treated with different levels of DAPT. Finally, the conclusions presented in this study are fully consistent with the previous studies and cannot be described as an isolated experimental artifact [9], [10], [14]–[16], [26]–[29], [35], [52], [54], [55].

## Materials and Methods

### Materials

DAPT (*N*-[*N*-(3,5-difluorophenacetyl)-L-alanyl]-*S*-phenylglycine *t*-butyl ester) was purchased from Calbiochem. HeLa S3 (Human Cervical Adenocarcinoma Cells ATCC Cat. No. CCL-2.2.) were maintained in Dulbecco's modified Eagle's medium (DMEM) supplemented with 10% fetal bovine serum. Lipofectamine™ LTX Reagent was from Life Technologies, Invitorgen (Cat. No. 15338-100). pSG5 vector (plasmid Stratagene, SV40 early promoter) carrying C99 sequences with 3xFLAG sequence at its C-terminus was described in the previous study [10]. Anti-flag αM2 monoclonal antibody was purchased from Sigma-Aldrich (cat. # F3165) and used as described in our previous study [10].

### Transfecting HeLa Cells

HeLa cells have been transfected using Lipofectamine LTX™ reagent following the manufacturer instructions. The transfection protocol and concentration of pSG5-cDNAwtC99 plasmid have been optimized to achieve desired assay sensitivity, a gradual increase in the substrate expression level, and a smooth transition from biphasic to the standard dose-response curves (Figs. 2–3). Initially concentration of pSG5-cDNAwtC99 plasmid have been optimized by western blots using anti-flag αM2 monoclonal antibody as described in our previous study [10]. Fine changes in concentration of pSG5-cDNAwtC99 plasmid have been optimized by the activity measurements as shown in figures 1,2,3. The cells were transfected in 24-well plates. The day before transfection the cells were trypsinized and counted. About 0.85×10^5^ cells were seeded per well in 0.5 ml of complete growth medium to achieve about 75% confluent cultures on the day of transfection. Before the transfection old medium was removed and replaced with fresh 0.5 ml of complete growth medium. For transfection 5X DNA concentration was diluted in 500 µl of Opti-MEM®I Reduced Serum Media and mixed with 0.7 µl of Lipofectamine LTX™ Reagent. The solution was gently mixed and incubated for 30 minutes at room temperature to form DNA- Lipofectamine LTX™ Reagent complexes. Following the 30 min incubation, 100 µl of the DNA- Lipofectamine LTX™ Reagent complexes was added directly to each well containing cells and mixed gently by rocking the plate back and forth. The cells were incubated at 37°C in a CO_2_ incubator for 18 hours.

### Secretion of Aβ1-40 in HeLa cells in the presence of increasing concentration of DAPT

Fresh medium was added 18 hours after the transfection (0.5 ml per well), to prepare the cells for incubation with DAPT [18], [51]. Different concentrations of DAPT were prepared in DMSO, and added to the cells so that the final DMSO concentration in the culture was 0.1% (v/v). DMSO vehicle represents 0 nM DAPT. The cells were incubated with DAPT at given concentrations for 15 hours.

### Sandwich ELISA for quantitative detection of Aβ 1–40

Sandwich ELISA kits for quantitative detection of human Aβ 1–40 peptides with highly selective monoclonal antibodies in a flexible 96 well format were purchased from Milipore (cat. #. TK40HS, The Genetics company Switzerland). The assay linear response is in the range from 6–125 pM of Aβ 1–40. The assays were performed by closely following the manufacturer instructions. To assure the most representative Aβ 1–40 samples, the samples were used immediately after collection following the manufacturer suggestion and our earlier reported experimental experiences [10]. Each well was filled with 50 µl of the antibody conjugate solution and 50 µl of sample. The Aβ 1–40 standards were supplied by the manufacturer and prepared in parallel with other samples. All of the prepared wells were wrapped in aluminum foil and incubated overnight at 4°C with gentle mixing. The next day each well was washed five times with 300 µl of wash solution. After each 20 minutes wash, the wash solution was poured out and the wells were dried by tapping the plates on an absorbing paper. Washed wells were filled with 100 µl of the enzyme conjugate solution, covered, and incubated for 30 min at room temperature with shaking. The washing procedure was repeated once again to remove excess of the enzyme-conjugate. Next 100 µl of the substrate solution was added in each well in dark, and kept for 30 minutes covered at room temperature. The reaction was quenched by adding 50 µl of stop solution to each well, and within 15 min the signal intensity was read by measuring absorption at 450 nm.

### Data Analysis

All experimental results were analyzed using MicroCal Origin 7.0 program, nonlinear regressions, and equations that represent specific mechanism. All results are reported as the best fit value ± standard error with two-sigma confidence intervals shown in square brackets (i.e. *x±y [p, q]*). Briefly, the standard error indicates random errors (i.e. precision) for each method [34], while the two sigma confidence intervals indicate the ability of given experimental setup to provide information about specific best-fit parameter [34]. The random error for presented experiments is low, as indicated by a low scatter from the best fit values. The sharp confidence intervals can be obtained by taking a large number of independent data points that cover all segments that define the measured functions [34].

The supplement section shows in detail derivation of the equation that can describe the biphasic activation-inhibition dose-response curves. The final form that was used in the nonlinear regression is:
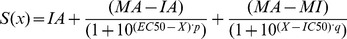
(1)where, *x* is a logarithmic value of the inhibitor concentration, *S(x)* is measured activity at inhibitor concentration *x*, *IA* is the activity at inhibitor concentration zero, *MA* is the calculated maximal activity, and *MI* is maximal inhibition. *EC50* and *IC50* represent activation and inhibition respectively, while *p* and *q* represent corresponding Hill's coefficients [34]. To facilitate numerical analysis the nonlinear regression was done with logarithmic values [34], and the best fit values were converted in units of concentration in the tables and graphs.

In similar fashion all standard dose-response curves were analyzed using equation [34]:
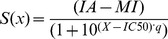
(2)The minimal steady-state equation that can describe how different enzyme-substrate and enzyme-inhibitor interactions can affect γ-secretase activity can be derived using connection matrix approach [74], computer program *Matematica* (Wolfram, Inc), and the corresponding reaction scheme (Fig. S1). The final minimal equation that contains only linear factors is:
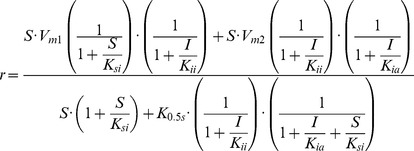
(3)


*S* and *I* represent substrate and inhibitor concentration respectively. *K_ia_* inhibitor activation constant, *K_ii_* inhibition constant, *K_0.5S_* substrate half-saturation constant (i.e. equivalent of Michaelis-Menten constant for the substrate), *K_is_* substrate inhibition constant, while *Vm1* and *Vm2* are the maximal turnover rates for the sub-saturated (complex 1 in Fig. 2) and saturated enzyme (complex 4 in Fig. 2).

## Supporting Information

Figure S1 **Schematic presentation of catalytic cycle of γ-secretase with multiple enzyme-substrate and enzyme-inhibitor interactions.** The mechanism is derived from the presented analysis of biphasic activation-inhibition dose-response curves. Free γ-secretase (E) can interact with substrate monomer (S) and produce catalytic complex (ES). Free enzyme can also interact with inhibitor (I), to produce IE complex which can interact with the substrate to form an “activating” IES complex. As third option, free enzyme can interact with multiple substrate molecules to form ESS catalytic complex. All three catalytic complexes can lead to catalysis, with different ability to produce different Aβ products as indicated in the earlier studies [10]. By choosing specific experimental conditions it is possible to gain specific insights about the different complexes. The catalytic complex ES dominates in absence of the inhibitor and at sub-saturating substrate. IES dominates at sub-saturating inhibitor and sub-saturating substrate. SES dominates in absence of the inhibitor and at saturating substrate. SESI and IEIS are catalytically inactive complexes that dominate at saturating inhibitor and saturating or sub-saturating substrate respectively.
(DOCX)

Figure S2 **Competitive inhibitors can regulate the extent of enzyme saturation with its substrate.** Competitive inhibitors of γ-secretase can be created by preparing compounds that can bind at the same time to the multiple sites of γ-secretase. First lead for such compounds can be head-to-tail, or side-by-side dimers, trimers of the currently known biphasic inhibitors as illustrated on the scheme. The lower panel shows how biphasic (thin red line) and competitive (green dashed line) inhibitors can affect the physiological response of γ-secretase to gradual increase in its substrate (tick black line). The biphasic inhibitors can induce saturation at otherwise sub-saturating substrate and decrease the maximal turnover rates, and thus drastically reduce γ-secretase capacity to process its substrates. The competitive inhibitors can only shift saturation to the higher substrate levels without changes in the enzyme's catalytic capacity [31]. The size of the shift depends on Michaelis-Menten constant for each product, and thus competitive inhibitors could have some capacity to modulate different products of γ-secretase. A possible drawback in application of competitive inhibitors could be still poorly understood shift to the longer more hydrophobic Aβ products that can be observed at the saturating substrate [10].
(DOCX)
