# Correction: Modulators of γ-Secretase Activity Can Facilitate the Toxic Side-Effects and Pathogenesis of Alzheimer's Disease

**DOI:** 10.1371/annotation/7cf2c08a-44d2-4db8-8584-d2e62e18d05d

**Published:** 2013-09-25

**Authors:** Željko M. Svedružić, Katarina Popović, Vesna Šendula-Jengić

A file was erroneously omitted from the list of Supporting Information. The full derivation of Equation 1 can be viewed here: 
