# Supplementary material for: Modulators of γ-Secretase Activity Can Facilitate the Toxic Side-Effects and Pathogenesis of Alzheimer's Disease
Source: PLoS One. 2013 Jan 7;8(1):e50759. doi: 10.1371/journal.pone.0050759 (PMC3538728; doi:10.1371/journal.pone.0050759)
Supplement: Figure S1 — Schematic presentation of catalytic cycle of γ-secretase with multiple enzyme-substrate and enzyme-inhibitor interactions. The mechanism is derived from the presented analysis of biphasic activation-inhibition dose-response curves. Free γ-secretase (E) can interact with substrate monomer (S) and produce catalytic complex (ES). Free enzyme can also interact with inhibitor (I), to produce IE complex which can interact with the substrate to form an “activating” IES complex. As third option, free enzyme can interact with multiple substrate molecules to form ESS catalytic complex. All three catalytic complexes can lead to catalysis, with different ability to produce different Aβ products as indicated in the earlier studies [10]. By choosing specific experimental conditions it is possible to gain specific insights about the different complexes. The catalytic complex ES dominates in absence of the inhibitor and at sub-saturating substrate. IES dominates at sub-saturating inhibitor and sub-saturating substrate. SES dominates in absence of the inhibitor and at saturating substrate. SESI and IEIS are catalytically inactive complexes that dominate at saturating inhibitor and saturating or sub-saturating substrate respectively. (DOCX) [file pone.0050759.s001.docx]

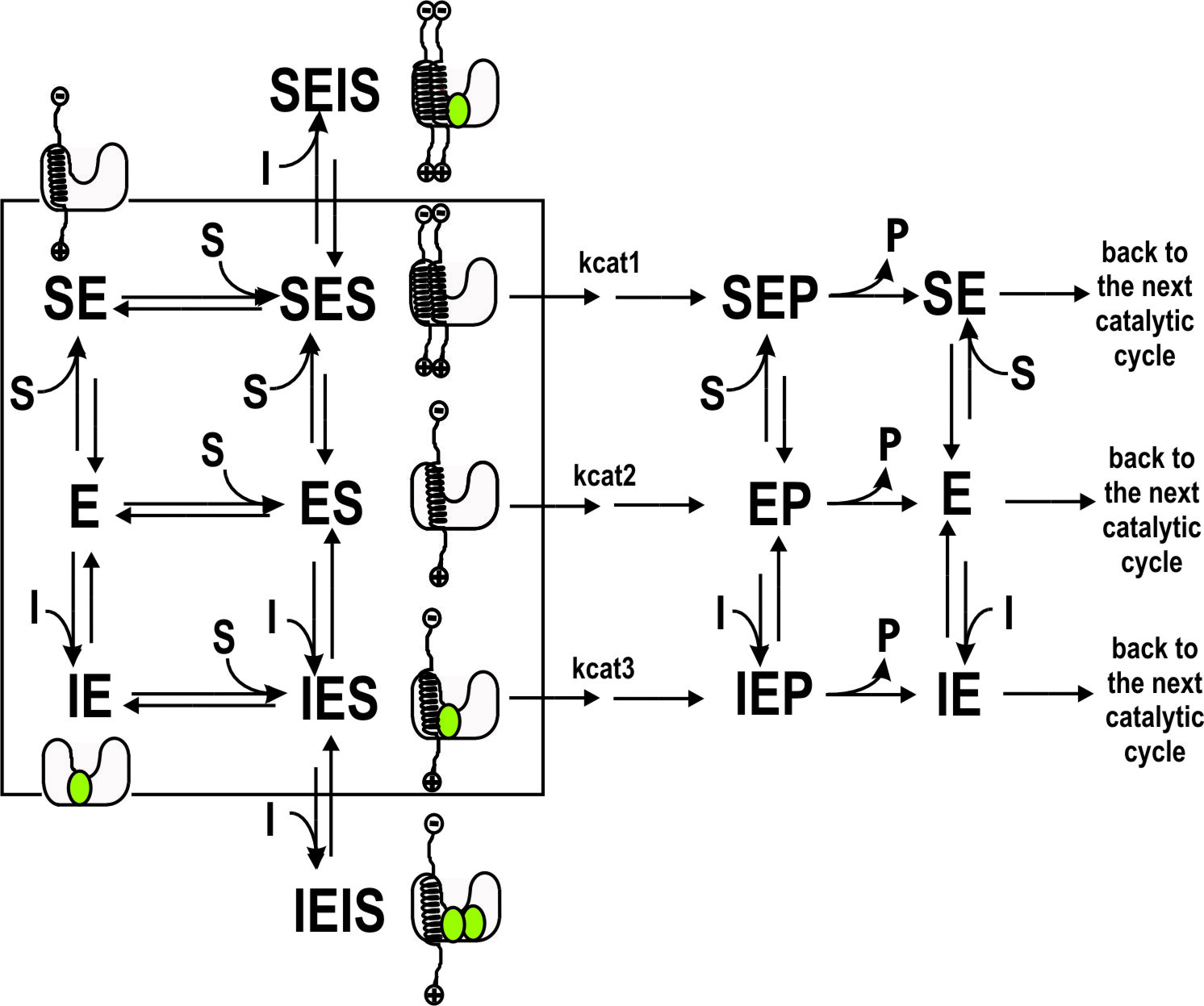


**Supplement Figure S1.** **Schematic presentation of catalytic cycle of γ-secretase with multiple enzyme-substrate and enzyme-inhibitor interactions**. The mechanism is derived from the presented analysis of biphasic activation-inhibition dose-response curves. Free γ-secretase (E) can interact with substrate monomer (S) and produce catalytic complex (ES). Free enzyme can also interact with inhibitor (I), to produce IE complex which can interact with the substrate to form an “activating” IES complex. As third option, free enzyme can interact with multiple substrate molecules to form ESS catalytic complex. All three catalytic complexes can lead to catalysis, with different ability to produce different Aβ products as indicated in our earlier studies.

By choosing specific experimental conditions it is possible to gain specific insights about the different complexes. The catalytic complex ES dominates in absence of the inhibitor and at sub-saturating substrate. IES dominates at sub-saturating inhibitor and sub-saturating substrate. SES dominates in absence of the inhibitor and at saturating substrate. SESI and IEIS are catalytically inactive complexes that dominate at saturating inhibitor and saturating or sub-saturating substrate respectively.
