# Supplementary material for: Modulators of γ-Secretase Activity Can Facilitate the Toxic Side-Effects and Pathogenesis of Alzheimer's Disease
Source: PLoS One. 2013 Jan 7;8(1):e50759. doi: 10.1371/journal.pone.0050759 (PMC3538728; doi:10.1371/journal.pone.0050759)
Supplement: Figure S2 — Competitive inhibitors can regulate the extent of enzyme saturation with its substrate. Competitive inhibitors of γ-secretase can be created by preparing compounds that can bind at the same time to the multiple sites of γ-secretase. First lead for such compounds can be head-to-tail, or side-by-side dimers, trimers of the currently known biphasic inhibitors as illustrated on the scheme. The lower panel shows how biphasic (thin red line) and competitive (green dashed line) inhibitors can affect the physiological response of γ-secretase to gradual increase in its substrate (tick black line). The biphasic inhibitors can induce saturation at otherwise sub-saturating substrate and decrease the maximal turnover rates, and thus drastically reduce γ-secretase capacity to process its substrates. The competitive inhibitors can only shift saturation to the higher substrate levels without changes in the enzyme's catalytic capacity [31]. The size of the shift depends on Michaelis-Menten constant for each product, and thus competitive inhibitors could have some capacity to modulate different products of γ-secretase. A possible drawback in application of competitive inhibitors could be still poorly understood shift to the longer more hydrophobic Aβ products that can be observed at the saturating substrate [10]. (DOCX) [file pone.0050759.s002.docx]

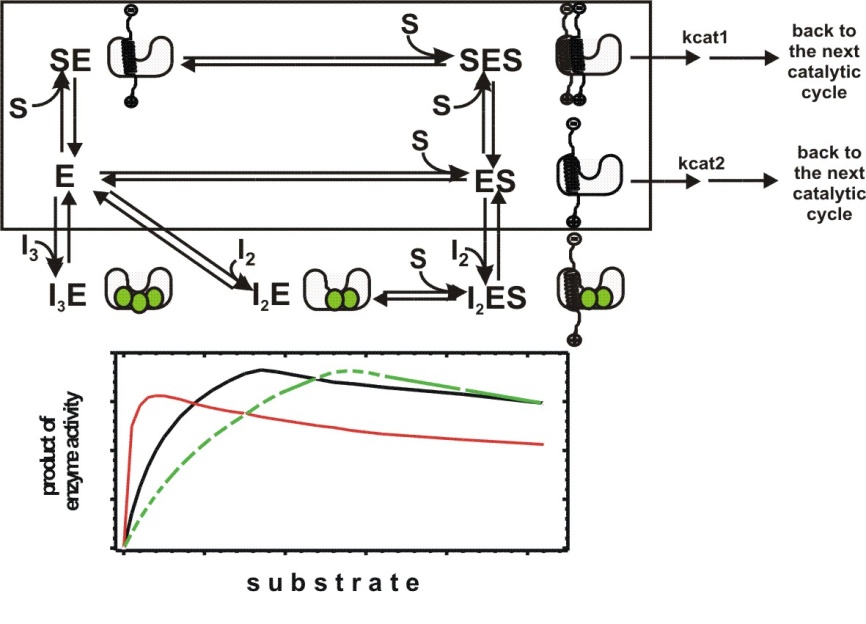


**Supplement Figure S2. Competitive inhibitors can regulate the extent of enzyme saturation with its substrate**. Competitive inhibitors of γ-secretase can be created by preparing compounds that can bind at the same time to the multiple sites of γ-secretase. First lead for such compounds can be head-to-tail, or side-by-side dimers, trimers of the currently known biphasic inhibitors as illustrated on the scheme.

The lower panel shows how biphasic (thin red line) and competitive (green dashed line) inhibitors can affect the physiological response of γ-secretase to gradual increase in its substrate (tick black line). The biphasic inhibitors can induce saturation at otherwise sub-saturating substrate and decrease the maximal turnover rates, and thus drastically reduce γ-secretase capacity to process its substrates. The competitive inhibitors can only shift saturation to the higher substrate levels without changes in the enzyme’s catalytic capacity. The size of the shift depends on Michaelis-Menten constant for each product, and thus competitive inhibitors could have some capacity to modulate different products of γ-secretase. A possible drawback in application of competitive inhibitors could be still poorly understood shift to the longer more hydrophobic Aβ products that can be observed at the saturating substrate.
