# Supplementary material for: Correction: Modulators of γ-Secretase Activity Can Facilitate the Toxic Side-Effects and Pathogenesis of Alzheimer's Disease
Source: PLoS One. 2013 Sep 25;8(9):10.1371/annotation/7cf2c08a-44d2-4db8-8584-d2e62e18d05d. doi: 10.1371/annotation/7cf2c08a-44d2-4db8-8584-d2e62e18d05d (PMC3783600; doi:10.1371/annotation/7cf2c08a-44d2-4db8-8584-d2e62e18d05d)
Supplement: Supplementary file 1 [file pone.7cf2c08a-44d2-4db8-8584-d2e62e18d05d.s001.doc]

Different equations, that describe different binding mechanisms, for multiple ligand molecules that can bind to different sites on one enzyme molecule have been described in the literature (1). For the reader’s convenience, here we provide detailed derivation of the equation that can describe the biphasic activation-inhibition dose-response curves. Different interactions can be described schematically:


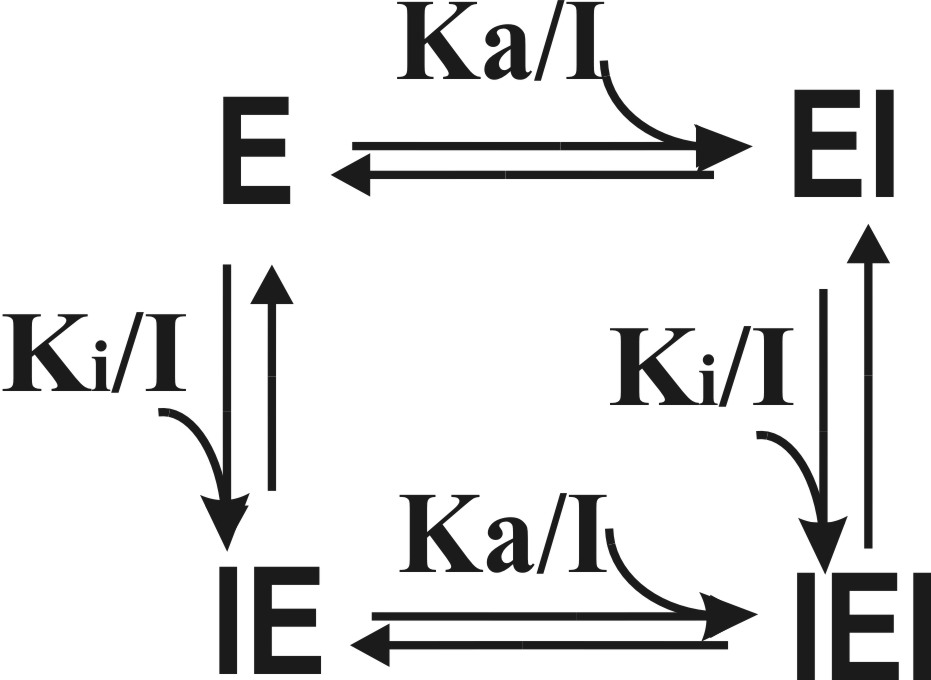


*E* is free enzyme concentration, *I* is free inhibitor concentration, *IE* and *EI* represent enzyme with inhibitor bound at the inhibition and the activation site respectively. *Ka* and *Ki* are dissociation constants for the activation site and inhibition sites respectively, while *IEI* represents the enzyme with inhibitor bound at the activation and the inhibition sites at the same time.

The relationship between different interactions and dissociation constants can be defined by equations:

$$K_{a}=\frac{I\cdot E}{EI}\Rightarrow\Rightarrow\Rightarrow EI=\frac{I\cdot E}{K_{a}}$$

$$K_{i}=\frac{I\cdot E}{IE}\Rightarrow\Rightarrow\Rightarrow IE=\frac{I\cdot E}{K_{i}}$$

$$K_{i}=\frac{I\cdot EI}{IEI}\Rightarrow\Rightarrow\Rightarrow IEI=\frac{I\cdot EI}{K_{i}}=\frac{I\cdot I\cdot E}{K_{i}\cdot K_{a}}$$

$$K_{a}=\frac{I\cdot IE}{IEI}\Rightarrow\Rightarrow\Rightarrow IEI=\frac{I\cdot IE}{K_{a}}=\frac{I\cdot I\cdot E}{K_{a}\cdot K_{i}}$$

Total enzyme concentration *Eo* is a sum of concentration for all of the different enzyme forms:

$$Eo=E+EI+IE+IEI=E+\frac{I\cdot E}{K_{a}}+\frac{I\cdot E}{K_{i}}+\frac{I\cdot I\cdot E}{K_{a}\cdot K_{i}}$$

Similarly total inhibitor concentration *Io* is a sum of free *I_F_* and bound inhibitor *I_B_* concentrations:

$$I_{0}=I_{F}+I_{B}=I_{F}+EI+IE+2\cdot IEI=I_{F}+\frac{I\cdot E}{K_{a}}+\frac{I\cdot E}{K_{i}}+2\cdot\frac{I\cdot I\cdot E}{K_{a}\cdot K_{i}}$$

The presented equation is derived taking in account that total inhibitor bound (*I_B_*) is equal to sum of all interacting enzyme forms, i.e. *EI*, *IE*, and *IEI* forms. *IEI* concentration is multiplied by factor 2 since two inhibitors are involved in formation of one *IEI* molecular complex.

Next we can define molar occupancy ratio, as the ratio between concentration of total inhibitor bound *(I_B_)* and the total enzyme concentration *(Eo)*:

$$r=\frac{I_{B}}{Eo}$$

Using the previously defined equations, we can write:

$$r=\frac{I_{B}}{Eo}=\frac{I_{o}-I_{F}}{Eo}=\frac{\frac{I\cdot E}{K_{a}}+\frac{I\cdot E}{K_{i}}+2\cdot\frac{I\cdot I\cdot E}{K_{a}\cdot K_{i}}}{E+\frac{I\cdot E}{K_{a}}+\frac{I\cdot E}{K_{i}}+\frac{I\cdot I\cdot E}{K_{a}\cdot K_{i}}}$$

Dividing both the numerator and the dominator with common factor *E*, we get:

$$\begin{matrix} r=\frac{\frac{I}{K_{a}}+\frac{I}{K_{i}}+2\cdot\frac{I\cdot I}{K_{a}\cdot K_{i}}}{1+\frac{I}{K_{a}}+\frac{I}{K_{i}}+\frac{I\cdot I}{K_{a}\cdot K_{i}}}=\frac{\frac{I}{K_{a}}+\frac{I\cdot I}{K_{a}\cdot K_{i}}+\frac{I}{K_{i}}+\frac{I\cdot I}{K_{a}\cdot K_{i}}}{\left(1+\frac{I}{K_{a}}\right)\cdot\left(1+\frac{I}{K_{i}}\right)}= \\ =\frac{\frac{I}{K_{a}}\cdot\left(1+\frac{I}{K_{i}}\right)+\frac{I}{K_{i}}\left(1+\frac{I}{K_{a}}\right)}{\left(1+\frac{I}{K_{a}}\right)\cdot\left(1+\frac{I}{K_{i}}\right)} \end{matrix}$$

few more simple algebra steps:

$$r=\frac{\frac{I}{K_{a}}\cdot\left(1+\frac{I}{K_{i}}\right)}{\left(1+\frac{I}{K_{a}}\right)\cdot\left(1+\frac{I}{K_{i}}\right)}+\frac{\frac{I}{K_{i}}\left(1+\frac{I}{K_{a}}\right)}{\left(1+\frac{I}{K_{a}}\right)\cdot\left(1+\frac{I}{K_{i}}\right)}=\frac{\frac{I}{K_{a}}}{\left(1+\frac{I}{K_{a}}\right)}+\frac{\frac{I}{K_{i}}}{\left(1+\frac{I}{K_{i}}\right)}$$

The most convenient final form can be obtained by multiplying the numerator and the denominator of each summands with *(Ka/I)* or *(Ki/I)* respectively:

$$r=\frac{1}{\left(1+\frac{K_{a}}{I}\right)}+\frac{1}{\left(1+\frac{K_{i}}{I}\right)}$$

The presented equation for molar occupancy is a sum of two summands, each represents one binding hyperbola which is asymptotically approaching value of 1. Depending on the *Ki*, *Ka*, and *I* values, each summands can vary between 0 and 1, and represents fractional occupancy of different inhibitor binding sites (i.e. when *I* is equal to *Ki*, fractional occupancy for the activation site is 0.5, or 50%). Since enzyme inhibition is usually analyzed using logarithmic graphs (2), we adapt presented equation to logarithmic graphs using well-known mathematical definition x = 10^Log x^ to get:

$$r=\frac{1}{\left(1+10^{\left(Log\frac{K_{a}}{I}\right)}\right)}+\frac{1}{\left(1+10^{\left(Log\frac{K_{i}}{I}\right)}\right)}$$

Knowing that *Log(a/b)* is equal to *Log(a)-Log(b)*:

$$r=\frac{1}{\left(1+10^{\left(LogK_{a}-LogI\right)}\right)}+\frac{1}{\left(1+10^{\left(LogK_{i}-LogI\right)}\right)}$$

In final step, the defined molar ratio occupancy is multiplied by the experimentally measured values, such as enzyme activity in the absence of the inhibitors *IA*, maximal activation *MA*, maximal inhibition *MI*, and finally the measured enzyme activity *S(i)* at inhibitor concentration *I(i)* :

$S(i)-IA=\frac{1}{\left(1+10^{\left(LogK_{a}-LogI\right)}\right)}\cdot(MA-IA)+\frac{1}{\left(1+10^{\left(LogK_{i}-LogI\right)}\right)}\cdot(MA-MI)$For example, using presented equation one can see that when the inhibitor concentration *I* is equal to *Ki*, the first factor will be 0.5 times (*MA*-*IA*), or 50% of maximal activation signal. To get the final form we will use some of the standard labels(2), *Ka* as *EC50*, *Ki* as *IC50,* and inhibitor concentration *I* as *X*:

$$S(x)=IA+\frac{(MA-IA)}{\left(1+10^{\left(EC50-X\right)\cdot p}\right)}+\frac{(MA-MI)}{\left(1+10^{\left(X-IC50\right)\cdot q}\right)}\cdot$$

Factors *p* and *q* represent Hill’s coefficients (1). These two factors are used in nonlinear regression as free fit parameters to estimate if the enzyme-inhibitor interaction follows simple one-to-one stoichiometry or some more complex mechanism should be considered (1). To avoid negative numbers in nonlinear regression, the first summand shows *EC50-X* and the second *X*-*IC50*, since the first represents the activation phase and the second inhibition phase (2).

1. Klotz IM (1997) Ligand-Receptor Energetics: A Guide for the Perplexed Wiley. 192 p.

2. Motulsky H, Christopoulos A (2004) Fitting Models to Biological Data Using Linear and Nonlinear Regression: A Practical Guide to Curve Fitting Oxford University Press, USA; 1 edition 352 p.
